# Supplementary material for: Transcriptome Analysis Reveals Vimentin-Induced Disruption of Cell–Cell Associations Augments Breast Cancer Cell Migration
Source: Cells. 2022 Dec 13;11(24):4035. doi: 10.3390/cells11244035 (PMC9776984; doi:10.3390/cells11244035)
Supplement: Supplementary file 1 [file cells-11-04035-s001.zip › DEGs and enrichment analysis.zip!/DEGs and enrichment analysis/WT_VIMvsVector GO enrichment/GSEA/WT_VIM/LENS_FIBER_CELL_DIFFERENTIATION/LENS_FIBER_CELL_DIFFERENTIATION(GO_0070306).html]

Details for gene set LENS\_FIBER\_CELL\_DIFFERENTIATION(GO:0070306)[GSEA]


|  || Dataset | fpkm.sample |
| Phenotype | sample.cls |
| Upregulated in class | WT\_VIM |
| GeneSet | LENS\_FIBER\_CELL\_DIFFERENTIATION(GO:0070306) |
| Enrichment Score (ES) | 0.9439658 |
| Normalized Enrichment Score (NES) | 1.4984703 |
| Nominal p-value | 0.0 |
| FDR q-value | 0.1629998 |
| FWER p-Value | 0.0 |
Table: GSEA Results Summary

  

Fig 1: Enrichment plot: LENS\_FIBER\_CELL\_DIFFERENTIATION(GO:0070306)
  
Profile of the Running ES Score & Positions of GeneSet Members on the Rank Ordered List

  

| PROBE | DESCRIPTION   (from dataset) | GENE SYMBOL | GENE\_TITLE | RANK IN GENE LIST | RANK METRIC SCORE | RUNNING ES | CORE ENRICHMENT || 1 | ENSG00000107859 | PITX3 |  |  | 641 | Infinity | -0.0108 | Yes |
| 2 | ENSG00000138083 | SIX3 |  |  | 1590 | Infinity | -0.0269 | Yes |
| 3 | ENSG00000026025 | VIM |  |  | 2388 | 1709.539 | 0.9440 | Yes |
| 4 | ENSG00000186790 | FOXE3 |  |  | 3327 | 3.955 | 0.9304 | No |
| 5 | ENSG00000140538 | NTRK3 |  |  | 3371 | 3.836 | 0.9319 | No |
| 6 | ENSG00000111186 | WNT5B |  |  | 3829 | 3.244 | 0.9260 | No |
| 7 | ENSG00000170819 | BFSP2 |  |  | 6181 | 1.992 | 0.8874 | No |
| 8 | ENSG00000105329 | TGFB1 |  |  | 8097 | 1.609 | 0.8559 | No |
| 9 | ENSG00000105325 | FZR1 |  |  | 8243 | 1.586 | 0.8543 | No |
| 10 | ENSG00000111276 | CDKN1B |  |  | 9723 | 1.392 | 0.8301 | No |
| 11 | ENSG00000188064 | WNT7B |  |  | 9943 | 1.366 | 0.8272 | No |
| 12 | ENSG00000186575 | NF2 |  |  | 12952 | 1.104 | 0.7770 | No |
| 13 | ENSG00000136842 | TMOD1 |  |  | 16244 | 0.903 | 0.7218 | No |
| 14 | ENSG00000142627 | EPHA2 |  |  | 16692 | 0.877 | 0.7147 | No |
| 15 | ENSG00000125875 | TBC1D20 |  |  | 17195 | 0.845 | 0.7067 | No |
| 16 | ENSG00000166225 | FRS2 |  |  | 17696 | 0.816 | 0.6987 | No |
| 17 | ENSG00000125864 | BFSP1 |  |  | 21341 | 0.617 | 0.6374 | No |
| 18 | ENSG00000151012 | SLC7A11 |  |  | 21597 | 0.602 | 0.6335 | No |
| 19 | ENSG00000117139 | KDM5B |  |  | 22622 | 0.535 | 0.6165 | No |
| 20 | ENSG00000196116 | TDRD7 |  |  | 23479 | 0.480 | 0.6023 | No |
| 21 | ENSG00000136603 | SKIL |  |  | 23480 | 0.480 | 0.6025 | No |
| 22 | ENSG00000178573 | MAF |  |  | 24185 | 0.422 | 0.5909 | No |
| 23 | ENSG00000166949 | SMAD3 |  |  | 24688 | 0.383 | 0.5826 | No |
| 24 | ENSG00000119608 | PROX2 |  |  | 26920 | 0.127 | 0.5449 | No |
| 25 | ENSG00000169554 | ZEB2 |  |  | 27993 | 0.000 | 0.5268 | No |
| 26 | ENSG00000154764 | WNT7A |  |  | 31003 | NaN | 0.4759 | No |
| 27 | ENSG00000118231 | CRYGD |  |  | 46052 | NaN | 0.2213 | No |
| 28 | ENSG00000182187 | CRYGB |  |  | 51074 | NaN | 0.1364 | No |
| 29 | ENSG00000117707 | PROX1 |  |  | 53003 | NaN | 0.1037 | No |
Table: GSEA details
[plain text format]

  

Fig 2: LENS\_FIBER\_CELL\_DIFFERENTIATION(GO:0070306)
  
Blue-Pink O' Gram in the Space of the Analyzed GeneSet

  

Fig 3: LENS\_FIBER\_CELL\_DIFFERENTIATION(GO:0070306): Random ES distribution
  
Gene set null distribution of ES for
 **LENS\_FIBER\_CELL\_DIFFERENTIATION(GO:0070306)**
